# DNA origami deposition on native and passivated molybdenum disulfide substrates

**DOI:** 10.3762/bjnano.5.58

**Published:** 2014-04-22

**Authors:** Xiaoning Zhang, Masudur Rahman, David Neff, Michael Louis Norton

**Affiliations:** 1 Department of Chemistry, Marshall University, One John Marshall Drive, Huntington, West Virginia 25755, United States

**Keywords:** atomic force microscopy (AFM), DNA origami, molybdenum disulfide (MoS_2_), pyrene, 1-pyrenemethylamine, surface modification

## Abstract

Maintaining the structural fidelity of DNA origami structures on substrates is a prerequisite for the successful fabrication of hybrid DNA origami/semiconductor-based biomedical sensor devices. Molybdenum disulfide (MoS_2_) is an ideal substrate for such future sensors due to its exceptional electrical, mechanical and structural properties. In this work, we performed the first investigations into the interaction of DNA origami with the MoS_2_ surface. In contrast to the structure-preserving interaction of DNA origami with mica, another atomically flat surface, it was observed that DNA origami structures rapidly lose their structural integrity upon interaction with MoS_2_. In a further series of studies, pyrene and 1-pyrenemethylamine, were evaluated as surface modifications which might mitigate this effect. While both species were found to form adsorption layers on MoS_2_ via physisorption, 1-pyrenemethylamine serves as a better protective agent and preserves the structures for significantly longer times. These findings will be beneficial for the fabrication of future DNA origami/MoS_2_ hybrid electronic structures.

## Introduction

Since it was first proposed and implemented by Rothmund in 2006 [1], DNA origami has offered a promising pathway for the construction of precisely programmed molecular architectures [2]. Through programmed, specific oligonucleotide recognition and hybridization, these DNA nanostructures can be used to combine, and therefore expand, the functional diversity of other materials [3]. The nanopatterning technologies of DNA origami structures allow for the lithographic transfer of a wide range of spatial information to other surfaces [3], enable the organized placement of nanoparticles [4] and receptors for the capture of proteins [5–6], and act as templates for the organization of carbon nanotubes [6–9]. This bottom-up process offers a tremendous advantage over photolithography, because is enables the patterning of surfaces with feature sizes less than 20 nm [10]. However, some materials may interfere with the base pairing responsible for origami structure formation and maintenance and are therefore unsuitable substrates for DNA origami deposition and patterning. For example, the folded structures are lost when they are deposited onto a graphene surface, because of π–π stacking between the single-stranded DNA and the graphene flakes [11]. In contrast, several materials have been found that enable the deposition of DNA origami structures while maintaining their structural integrity. These materials include mica [12], silicon dioxide [13], gold [14], and graphene oxide [2]. The ideal substrate surface must be atomically smooth to enable optimal patterning and imaging through atomic force microscopy (AFM) because the origami structures are very thin and conformal. A final substrate property that needs to be considered for maximal utility is that the material should possess conductive or semiconductive electronic properties, so as to enable complex and diverse circuit designs, thereby providing functionality essential for the construction of sensing biodevices with extraordinary sensitivity, rapid readout and good stability.

As a layered two-dimensional (2D) material, molybdenum disulfide (MoS_2_) exhibits robust mechanical properties and superior electrical performance [15]. Compared to the zero bandgap of graphene, the bandgap of MoS_2_ is adjustable. With decreasing the thickness, the gap progressively shifts from 1.29 eV to over 1.90 eV [16], which makes it a promising material for transistor, optoelectronic and energy harvesting applications [17]. Compared to conventional semiconductor materials such as silicon, MoS_2_ is readily processed as a prototypical nanomaterial. MoS_2_ nanosheets, nanofibers, and nanorods have been prepared [15], which means the material could readily be used to construct electronic devices with nanoscale dimensions. Several recent studies have examined the interaction of DNA with MoS_2_ [15,18]. However, the adsorption of DNA origami structures on MoS_2_ surfaces has not previously been explored. The behavior of DNA origami structures on this "S–Mo–S" sandwich structured compound is reported below for the first time. An unanticipated observation was that DNA origami structures decompose on contacting the MoS_2_ surface. However, the shape of DNA origami constructs can be preserved with the aid of an adhesion layer composed of either pyrene or 1-pyrenemethylamine. It is expected that this method will be helpful in the development of future applications for the DNA origami/MoS_2_ hybrid system in nanoelectronics, optoelectronics and sensing.

## Results and Discussion

**Figure 1:**
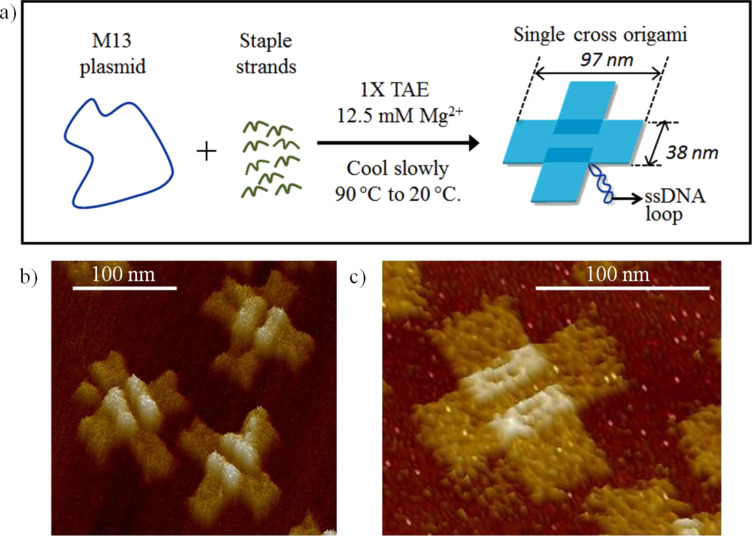
Schematic illustration representing (a) self-assembly of M13 plasmid (dark blue) and staple strands (deep green) to form cross shaped DNA origami (light blue); (b) and (c) represent AFM 3D images of formed DNA origami on mica. Each of the tiles forms the cross-like structure shown in (a). The 3D images of the cross-shaped DNA origami emphasize the overlap of the two domains.

Cross-like DNA origami structures were first constructed by using the protocols of Liu [19]. A schematic representation of such a tile is shown in Figure 1a. The key feature of this DNA origami structure is that the tile is composed of two rectangular domains (97 nm × 38 nm for each domain), one stacked above the other. Further experimental details are included in Supporting Information File 1. High-resolution AFM images of the cross-like DNA origami structure on a mica surface are shown in Figure 1b and Figure 1c.

**Figure 2:**
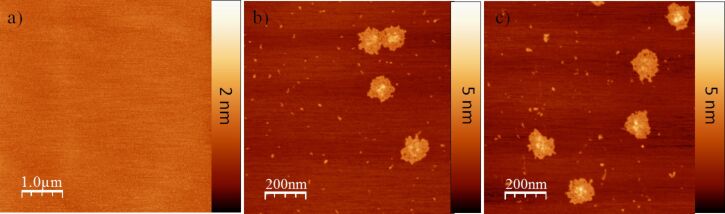
Representative AFM images of the pristine MoS_2_ substrate before (a) and after the DNA origami solution was deposited onto it with different incubation times: 10 s (b) and 30 s (c).

The surface morphology and surface roughness of the MoS_2_ mineral sample were investigated by using AFM. As anticipated, the newly cleaved pristine MoS_2_ surface was very smooth, featureless, and homogenous (Figure 2a). For optimal AFM imaging, the roughness of the surface should be kept as low as possible in order to avoid additional noise in the imaging of these very thin (about 2 nm) objects. Based on measurements taken over a 5 μm × 5 μm area similar to that shown in Figure 2a, the root mean square roughness (RMS) of the MoS_2_ surface was found to be 0.92 Å, indicating that MoS_2_ presents an ideal physical surface for the deposition of flat DNA nanostructures.

Importantly, AFM imaging reveals that shape and structure of the DNA origami constructs tended to be lost (Figure 2b) when the DNA origami was incubated on the MoS_2_ surface for only 10 s. This observation suggests that the complementary DNA double strands inside the origami structures are denatured due to the interaction with the MoS_2_ surface. The deposition time was also expanded to 30 s in order to gain some level of insight into the kinetics of the surface-driven denaturation reaction. Comparison with the images resulting from 30 s of incubation, shown in Figure 2c, suggests that the denaturation process appears to be complete in 10 s, since no significant changes with regard to the morphology of the DNA origami were observed. Although the extra staples present in the synthesis solution were removed via dialysis, debris is observed in the background of these images and is attributed to small quantities of additional staple DNA released from the DNA origami structures. It may be observed that these ssDNA staples adsorbed to the pristine MoS_2_ surface adopt many different structures, most likely originating partially from intra-strand base pairing and partially from the strong interaction between DNA bases and the MoS_2_ surface [11].

Recent studies indicate that the MoS_2_ surfaces have high polarity and hydrophilicity [20], which lead DNA to adsorb through van der Waals forces between the four nitrogenous nucleobases and the basal plane of MoS_2_ [18]. For example, in the report of Maddocks et al. [21], guanine, one of the four DNA bases, was observed, by using scanning tunneling microscopy (STM), to form a stable two-dimensional ordered array. These results are of crucial importance, as they support the hypothesis that the van der Waals interaction between MoS_2_ and the DNA in the origami is of sufficient strength to destabilize the hydrogen bonds as well as the π–π stacking interactions in the relatively short duplex regions within the DNA origami constructs. This leads to denaturation of these complexes. The transition from double-stranded DNA to single-stranded DNA would be expected to require an expansion of the size (footprint) of the origami, however if the interaction between the bases of the DNA origami structure and the MoS_2_ substrate are of sufficient strength, further dispersion/equilibration in two dimensions would not be anticipated. This is consistent with the observation that the structures do not evolve significantly between 10 and 30 s of incubation.

### Surface modification using 1-pyrenemethylamine and pyrene

**Figure 3:**
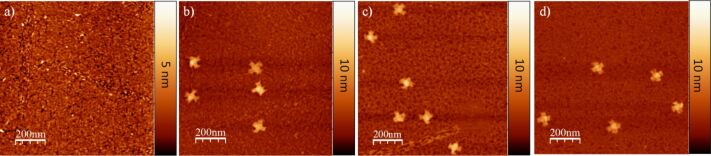
(a) AFM image of the MoS_2_ basal plane after exposure to 0.5 mM 1-pyrenemethylamine methanol solution for 5 min. (b) DNA origami with a cross shape imaged immediately after deposition onto pre-modified MoS_2_ substrates. (c) AFM images of DNA origami adsorbed on the MoS_2_ after 24 h and (d) 48 h.

1-pyrenemethylamine has been employed as a linker to bind DNA to graphene and carbon nanotube surfaces [3,22]. Here, we adopted a similar approach by treating the MoS_2_ surface with 1-pyrenemethylamine, a bifunctional bridging compound, and then exposing the substrate to a solution of DNA origami constructs. While the surface roughness increased significantly to 5.3 Å after surface modification (Figure 3a), we found that DNA nanostructures remained intact in the presence of the 1-pyrenemethylamine adhesion layer (Figure 3b–d), in contrast to the DNA nanostructures deposited on the bare MoS_2_ surface. This is readily understood in the context of a model, in which the pyrenyl group in 1-pyrenemethylamine is bound to the highly planar, polar, and polarizable MoS_2_ surface by van der Waals forces and forms an adhesion layer. Conversely, the amine group in 1-pyrenemethylamine interacts electrostatically with the phosphate group of the DNA origami constructs, binding them to the surface through formation of salt bridges. Pre-adsorption of 1-pyrenemethylamine molecules serves to mask the MoS_2_ surface and to sufficiently reduce the van der Waals interaction between MoS_2_ and the double stranded DNA in the origami constructs, thereby preserving their original structure.

It is known that MoS_2_ readily adsorbs water molecules from the atmosphere [20]. Because DNA origami structures are unstable and easily degraded in a pure H_2_O environment [11], it was necessary to perform a variable-time study to investigate any morphological changes in the DNA origami structure on the modified MoS_2_ surface in ambient environment. No significant changes were noted after 24 h and 48 h, respectively (Figure 3c and Figure 3d), indicating that the 1-pyrenemethylamine layer does not experience significant water accumulation from the atmosphere. The morphology after 120 h was also studied (see Supporting Information File 1, Figure S2). AFM imaging indicated a good retention of the structure of the DNA origami constructs. This relatively stability over a relatively long time is favorable for future construction of DNA origami-based MoS_2_ sensing devices.

**Figure 4:**
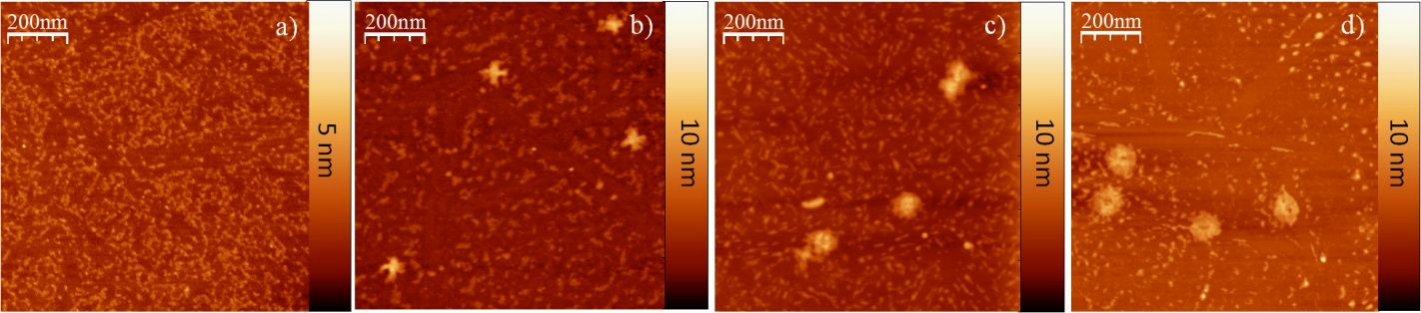
(a) MoS_2_ substrate incubated with a 0.5 mM pyrene–methanol solution for 5 min. (b) Cross-shaped DNA origami nanostructures deposited on MoS_2_ pre-treated with pyrene and immediately imaged by AFM in air. The AFM images were also recorded after 24 h (c) and 48 h (d). The observed DNA origami structure degrades over time.

Since the pyrene moiety, a primary functional component group of 1-pyrenemethylamine, is known to interact with MoS_2_ as an intercalant [23], pyrene was also studied in this research. Using the same conditions for surface film fabrication, a MoS_2_ substrate was dipped into a pyrene–methanol solution, followed by the deposition of DNA origami constructs onto the treated substrate. Apparently, the surface coverage of pyrene on the MoS_2_ was not as smooth as that of 1-pyrenemethylamine (Figure 4a), which might be partially caused by the lower polarity of the pyrene molecules. Although initial images (Figure 4b) indicated a retention of the origami structures, AFM images of DNA origami constructs deposited on the pyrene-modified MoS_2_ surface recorded at 24 h (Figure 4c) and 48 h (Figure 4d) after deposition demonstrated a progressive decomposition of the DNA origami structures.

Although this might be attributed to the accumulation of H_2_O molecules on the MoS_2_ surface caused by the limited surface coverage of pyrene, other mechanisms for disruption of the structure, including the strong van der Waals interactions with pyrene or even pyrene intercalation into the DNA [24–27], may be active. Additionally, a control experiment was performed to confirm that methanol, or a methanol impurity, was not possibly contributing to the preservation of the DNA origami structures (see Supporting Information File 1 for full experimental data). In summary, although both 1-pyrenemethylamine and pyrene can prevent immediate DNA origami structural disruption caused by interaction with the MoS_2_ substrate, the protective effect of the 1-pyrenemethylamine surface layer is much greater than that of pyrene.

## Conclusion

MoS_2_ has a great potential as a transducer material in future biosensor applications. In this work, the behavior of DNA origami structures on a MoS_2_ surface was studied for the first time. Our results revealed that DNA origami nanostructures are not stable when in direct contact with the MoS_2_ surface. This can be attributed to the van der Waals interaction between nucleobases and the basal plane of MoS_2_, which destabilizes the double-stranded structure of the DNA origami constructs. However, it was found that DNA origami structures retain their structures for a relatively long time when adsorbed onto MoS_2_ surfaces that have first been treated to generate a 1-pyrenemethylamine surface adhesion layer. The structure-preserving properties of a pyrene protective layer were compared with those of a 1-pyrenemethylamine layer. It was found that 1-pyrenemethylamine provides much better protection of the DNA origami structure than the pyrene layer. Although the microscopic mechanism was not determined in this work, it is possible that the methyl spacer in 1-pyrenemethylamine is sufficient to disrupt the van der Waals interaction between pyrene and DNA, which enables the origami to retain its base complementarity and therefore its shape. This model, in which the pyrenyl moiety of the 1-pyrenemethylamine molecule enables it to physisorb on to the MoS_2_ surface while the amine functionality enables the electrostatic tethering of the 1-pyrenemethylamine to the DNA, is consistent with the reported intercalation of pyrene into MoS_2_ [23] and the known use of amines to efficiently bind DNA to surfaces [23]. This method will benefit research involving biomolecular sensing on MoS_2_ in general, and the use of DNA origami to generate nanostructures on MoS_2_ surfaces specifically.

## Experimental

MoS_2_ was obtained from Ward’s (Rochester, N.Y.). Methanol, pyrene, and 1-pyrenemethylamine hydrochloride were obtained from Sigma-Aldrich. We used M13mp18 ssDNA plasmid (7249 bp) and short complementary DNA staple strands to program the cross-shaped DNA origami (the details are provided in Supporting Information File 1). To remove the excess staple strands, the DNA origami solutions were dialyzed with Amicon Ultra Centrifugal Filter Devices (100,000 molecular weight cutoff) for 30 min; 1 × TAE with 12.5 mM Mg^2+^ was used as the buffer solution. After dialysis, the solution of DNA origami structures was recovered from the dialysis tubing and prepared for imaging. M13mp18 single stranded phage DNA was purchased from Bayou Bio-Labs, while the short DNA staple strands were purchased from Integrated DNA Technologies (IDT). DI water (Millipore, 18 MΩ·cm) was used to prepare all buffer solutions.

To deposit DNA nanostructures onto the modified MoS_2_, freshly cleaved MoS_2_ samples were first dipped into 0.5 mM 1-pyrenemethylamine/pyrene–methanol solution for 5 min, then washed with 400 μL of pure methanol and gently dried in an argon stream. Subsequently, 5 μL of dialyzed DNA origami in 1 × TAE/12.5 mM MgCl_2_ buffer was dispensed on the top of the treated MoS_2_ surface. Ten seconds later, the DNA drop was blown dry with an argon stream, and then washed with 400 μL of Milli-Q water in order to remove excess salts from the surface. Next, the morphologies of the DNA origami on the substrates were determined by using a Bruker Multimode AFM with Nanoscope VI controller in SCANASYST-AIR mode. All steps were performed at room temperature. All AFM images were processed and rendered by using the software WSxM [28].

## Supporting Information

Supporting Information features additional information about the formation of self-assembled DNA origami nanostructures and a study of the effect of methanol on the preservation of DNA origami structures.

File 1 Additional experimental data.
