# Supplementary material for: DNA origami deposition on native and passivated molybdenum disulfide substrates
Source: Beilstein J Nanotechnol. 2014 Apr 22;5:501–6. doi: 10.3762/bjnano.5.58 (PMC7879407; doi:10.3762/bjnano.5.58)
Supplement: File 1 — Additional experimental data. [file Beilstein_J_Nanotechnol-05-501-s001.pdf]

Supporting Information

for

DNA origami deposition on native and passivated molybdenum disulfide substrates

Xiaoning Zhang, Masudur Rahman, David Neff and Michael L. Norton *

Address: Department of Chemistry, Marshall University, One John Marshall Drive, Huntington, West Virginia 25755, United States

Email: Michael L. Norton* - norton@marshall.edu

* Corresponding author

Additional experimental data

Self-assembled DNA origami nanostructure formation

The detailed procedure for the formation of DNA origami is as follows: A mixture of staple strand DNA containing the same concentration of each staple was prepared first. This pre-mix was then added into a solution of single-stranded M13mp18 phage DNA (M13) and TAE buffer containing Mg^{2+} ions, and brought to a final volume of 50 μL with DI water. The final solution mixture contains 50 nM of each staple strand, 10 nM M13, 40 mM Tris, 20 mM sodium acetate, 1 mM EDTA, and 12.5 mM magnesium acetate (1 × TAE buffer with Mg^{2+}). The solution was then heated to 90 °C in a thermal cycler (MWG AG Biotech Primus 96 Plus) and allowed to cool slowly (for approximately 13 h) to 20 °C. The produced solution contained self-assembled DNA origami suspended in TAE buffer.

Study of the effect of methanol on the preservation of DNA origami structures

In order to evaluate the possibility that the passivation layer could result from an impurity in the solvent, methanol, a control experiment was conducted. Maintaining the same experimental conditions for this control experiment as for the chemical modification protocol, pristine MoS_2 was first dipped into pure methanol for 5 min, and then a dialyzed DNA Origami solution was applied to the treated MoS_2 surface. AFM imaging of this MoS_2 surface after the deposition of DNA origami (Figure S1b) indicated that the DNA origami constructs on the MoS_2 surface pre-treated with methanol had lost their folded structure. This result further supports the suggestion that the preservation of the DNA origami folded structure is due to the 1-pyrenemethylamine or pyrene coatings.

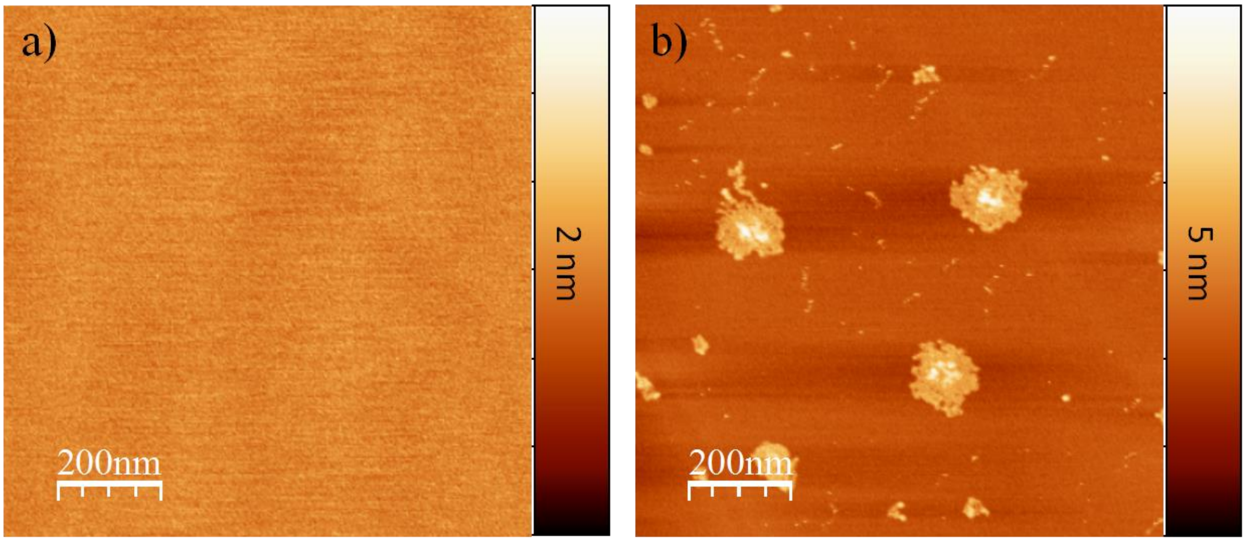

Figure S1: AFM image of (a) the basal plane of MoS_2 after exposure to methanol. (b) Cross-shaped DNA Origami adsorbed on a methanol pre-treated MoS_2 surface.

Morphology of DNA origami on 1-pyrenemethylamine passivated MoS_2 after 120 hours

Figure S2 presents an AFM image of DNA Origami deposited on the 1-pyrenemethylamine modified MoS_2 surface recorded at 120 hours after deposition, demonstrating that the cross-like origami structures persist for significant amounts of time on this surface.

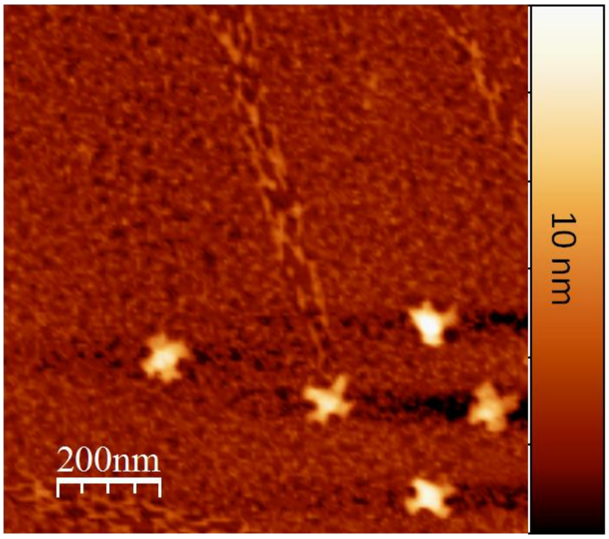

Figure S2: AFM image of DNA Origami adsorbed on 1-pyrenemethylamine passivated MoS_2 surface after 120 h under ambient atmospheric conditions.
